# COVID-19: Opportunities for interdisciplinary research to improve care for older people in Sweden

**DOI:** 10.1177/1403494820969544

**Published:** 2020-11-08

**Authors:** Rebecca Baxter, Wossenseged Birhane Jemberie, Xia Li, Mahwish Naseer, Mascha Pauelsen, Jacques Shebehe, Emilia W.E. Viklund, Xin Xia, Linn Elena Zulka, Andreea Badache

**Affiliations:** 1 Department of Nursing, Umeå University, Sweden; 2 Department of Social Work, Umeå University, Sweden; 3 Centre for Demographic and Ageing Research (CEDAR), Umeå University, Sweden; 4 Department of Medical Epidemiology and Biostatistics, Karolinska Institutet, Sweden; 5 School of Education, Health and Social Studies, Dalarna University, Sweden; 6 Ageing Research Centre, Department of Neurobiology, Care Sciences and Society, Karolinska Institutet, Sweden; 7 Department of Health Sciences, Luleå University of Technology, Sweden; 8 Clinical Epidemiology and Biostatistics, School of Medical Sciences, Örebro University, Sweden; 9 Department of Health Sciences, Faculty of Education and Welfare Studies, Åbo Akademi University, Finland; 10 Department of Neurobiology, Care Sciences and Society, Karolinska Institute, Sweden; 11 Department of Psychology, Centre for Ageing and Health (AgeCap), University of Gothenburg, Sweden; 12 Department of Disability Sciences, School of Health Sciences, Örebro University, Sweden; 13 Swedish Institute of Disability Research, Örebro University, Sweden

**Keywords:** COVID-19, Sweden, aged, ageing, older people, public health, patient and public engagement (PPE), evidence-based policy

## Abstract

The emergence of COVID-19 has changed the world as we know it, arguably none more so than for older people. In Sweden, the majority of COVID-19-related fatalities have been among people aged ⩾70 years, many of whom were receiving health and social care services. The pandemic has illuminated aspects within the care continuum requiring evaluative research, such as decision-making processes, the structure and organisation of care, and interventions within the complex public-health system. This short communication highlights several key areas for future interdisciplinary and multi-sectorial collaboration to improve health and social care services in Sweden. It also underlines that a valid, reliable and experiential evidence base is the sine qua non for evaluative research and effective public-health systems.

## Introduction

This short communication aims to highlight opportunities for interdisciplinary research and multi-sectorial collaboration, with a view towards strengthening health and social care services for older people living in Sweden in the wake of the coronavirus disease 2019 (COVID-19) pandemic. In this discussion, we emphasise the need for research to evaluate decision-making processes and interventions across various sectors, the structure and organisation of the health-care system and the impact of COVID-19 on the lived experience of older people in Sweden. The authors are doctoral affiliates of the Swedish National Graduate School for Competitive Science on Ageing and Health (SWEAH). SWEAH’s mission is to support interdisciplinary research to improve the quality of life, well-being and health outcomes for the ageing population.

As of 29 June 2020, the Swedish National Board of Health and Welfare (Socialstyrelsen) reported that people aged ⩾70 years accounted for 90% (*n*=4674) of all COVID-19-related deaths (*n*=5194) [1]. Of those aged ⩾70 years who died, 26.9% were receiving social services and 51.5% were residents in long-term care (LTC) facilities [1]. In mid-March 2020, the Public Health Agency of Sweden (Folkhälsomyndigheten) recommended self-isolation and physical/social distancing for people aged ⩾70 years [2], and in late March 2020, the Swedish government passed legislation restricting non-essential visitors to LTC facilities and special accommodation for older people [3]. However, many older people rely on outside help from informal caregivers, and around 71% of people aged ⩾65 years living in ordinary housing use municipal home-care services [4], making ‘perfect’ adherence to distancing regulations almost impossible. For example, it has been estimated that over a 2-week period, a person using home-care services meets an average of 15 health and social care workers, each of whom have contact with more than 10 clients [4]. This is concerning, as numerous care transitions and contact with different service providers have been associated with an increased risk of infection for older people [5], illustrating the complexity of integrating safety regulations and care requirements across multiple sectors.

Additional barriers to maintaining standards of care include resource shortages resulting from cuts to health and social care funding [6], unstable working conditions [7,8] and casual employment contracts [8,9]. Surveillance and preparedness are central to enacting appropriate responses to manage the spread of infections [10,11]. Yet, basic education and training regarding hygiene routines and infection control measures were already lacking prior to COVID-19 [7,9,12]. Data collected between the 9th and 22nd of May 2020 regarding Swedish LTC workers’ compliance with processes related to basic hygiene routines and dress codes found only 59% adherence compared to 51% in 2019 [13]. Service providers reported lacking basic personal protective equipment, and many employees continued to work due to being short-staffed and feeling insecure about their employment [14,15]. Interdisciplinary and multi-sectorial collaboration is required to create better working conditions for staff and improve safety for people receiving homecare services or living in LTC. This should encompass provision of education and training for health and social care employees, implementation of user-informed feedback loops to ensure responsive practices to emerging data, development of evidence-based clinical strategies for infection prevention and control and operationalisation of longitudinal surveillance involving multiple data sources.

Much of the responsibility for these pandemic-mitigating measures was placed on individuals to restrict contact with their families and communities, which is worrying, given that about 36% of adults aged ⩾65 years in Sweden live alone [16,17] and experience loneliness and social isolation [18]. To maintain social connections and alleviate feelings of loneliness, many people around the world have turned to digital tools, which has actualised the importance of digital competence. Opportunities have arisen for new and existing digital technology to monitor medication adherence, enhance psychosocial well-being and facilitate connections between older people and their families, communities and healthcare services [19–22]. Research suggests that older people are positive towards digital technology, especially if it is perceived as beneficial and user-friendly [23]. However, such digital interventions might worsen health inequities given that 31% of Swedes aged >75 years did not use the Internet in 2019 [24], and poor accessibility and mistrust in information technology systems can affect adherence to digital services [25]. It is therefore important to evaluate the experience and evidence base for digital services carefully before implementing them into routine post-pandemic practice. For example, increased use of digital telehealth services may be necessary in the short term, but in the long term, this may risk opportunities for some older people to communicate and build relationships with care providers [26,27]. Inviting older people to share their experiences and preferences regarding the use of digital tools could contribute towards better implementation of effective and innovative interventions in health and social care services.

Experts in the field of ageing science have criticised the absence of targeted measures and adequate staffing to support frail older people living in ordinary homes and LTC facilities [28]. The Swedish Pensioners’ Association also recently criticised the Public Health Agency of Sweden and the Swedish Civil Contingencies Agency for not including the opinion of people ⩾80 years in their survey measuring the effect of the pandemic on care and services for older people [29], leading the agencies to acknowledge their error [30]. Research to evaluate decision-making processes, interventions and outcomes should therefore strive to encompass the perspective of multiple stakeholders. Researchers, healthcare professionals and policymakers now have the *opportunity* and *responsibility* to partner with older people to set priorities for the development of streamlined and integrative public-health systems.

## Opportunities for interdisciplinary research

Identification and evaluation of indicators for follow-up is necessary during and after emergency situations to improve future decision-making processes and public-health system preparedness, responsiveness and resilience. As SWEAH-affiliated doctoral researchers with diverse academic and professional backgrounds, we point to five key areas that require interdisciplinary and multi-sectorial research and collaboration to strengthen current and future health and social care services for older people:

- Enabling shared decision making and streamlined information sharing between branches of health and social care services to provide safe and integrated care;
- Improving working conditions for health and social care professionals via provision of adequate resources, job security and training for future healthcare crises through simulation and embedded education;
- Implementing user-informed feedback loops to ensure that policies and interventions are appropriate and responsive to the needs of health and social care systems and the people who use them;
- Cultivating opportunities for alternative forms of connection and communication for older people, including evidence-based digital technology;
- Creating opportunities for partnership with older people to set priorities and co-design research and policy that directly affects their lived experience and everyday lives.

## Conclusion

We acknowledge that this communiqué is far from comprehensive. However, it is our hope that the lives of older people in Sweden can be improved through interdisciplinary and multi-sectorial collaboration among health and social care systems. Shared governance must encompass the lived experience of both healthcare providers and older people to improve outcomes across the care continuum. Emerging from this pandemic, we must be vigilant that the measures taken during this time do not become standard practice without support from a valid, reliable and *experiential* evidence base.
